# A Comparative Study on Distancing, Mask and Vaccine Adoption Rates from Global Twitter Trends

**DOI:** 10.3390/healthcare9050488

**Published:** 2021-04-21

**Authors:** Satyaki Roy, Preetam Ghosh

**Affiliations:** 1 Department of Genetics, University of North Carolina, Chapel Hill, NC 27599, USA; 2 Department of Computer Science, Virginia Commonwealth University, Richmond, VA 23284, USA; pghosh@vcu.edu

**Keywords:** COVID-19, machine learning, tweets, sentiment analysis, adoption rates, socioeconomic

## Abstract

COVID-19 is a global health emergency that has fundamentally altered human life. Public perception about COVID-19 greatly informs public policymaking and charts the course of present and future mitigation strategies. Existing approaches to gain insights into the evolving nature of public opinion has led to the application of natural language processing on public interaction data acquired from online surveys and social media. In this work, we apply supervised and unsupervised machine learning approaches on global Twitter data to learn the opinions about adoption of mitigation strategies such as social distancing, masks, and vaccination, as well as the effect of socioeconomic, demographic, political, and epidemiological features on perceptions. Our study reveals the uniform polarity in public sentiment on the basis of spatial proximity or COVID-19 infection rates. We show the reservation about the adoption of social distancing and vaccination across the world and also quantify the influence of airport traffic, homelessness, followed by old age and race on sentiment of netizens within the US.

## 1. Introduction

The course and long-term impact of COVID-19 on human life will be determined largely by public attitude [1]. Since its inception in 2020, COVID-19 has left an indelible imprint on human existence and laid bare the socioeconomic, political, and attitudinal limitations of modern man [2]. COVID-19 has attracted the unprecedented attention of the international leadership because it has adversely affected both developed and developing nations alike, threatening to undo the years of sustainable growth especially in the least developed nations [3]. Epidemiologists agree that the adverse effects of future outbreaks can be contained by encouraging behavioral changes on the part of governments and individuals [4].

In the early stages of research on development, clinical trials and distribution of large-scale immunization measures, and social distancing continues to be a reliable strategy to curb infection spread and save lives [5]. The downside of the lockdown and international travel bans is the impending economic downturn and stock market decline [6]. This all-round existential threat prompted the scientific community constituting epidemiologists, sociologists, medical practitioners, and computer scientists to study the different aspects of the pandemic [7]. Several machine learning (ML) models are being created using the available clinical and epidemiological information to predict the course of the pandemic [8]. Specifically, ML models built around clinical data [8] are predicting the at-risk population [9,10], while epidemiological ML models are estimating the dynamics of contagion spread. Existing mechanistic, curve-fitting models [11], and supervised and unsupervised ML models [12] are bringing new insights into COVID spread dynamics. Khan et al. performed regression analysis, cluster analysis, and principal component analysis on Worldometer infection count data to study how variation in large-scale testing affects infection counts [13]. Roy et al. utilized regression studies to report pre-lockdown factors that impact post-lockdown contagion [14] and topic modeling to find the least and most affected economic sectors in the US [15]. They also proposed dynamic lockdown and mobility management strategies to curb contagion on the basis of economic and epidemiological profiles [16,17].

The sentiment about the COVID-19 mitigation will determine the strategies of effective policies and regulations to curb new waves or future outbreaks. Geldsetzer created a knowledge-base of perception of 80,000 volunteers from the US and UK, by performing an online survey [18]. Samuel et al. showed that Naive Bayes can achieve high sentiment prediction on COVID related tweets [19]. They outlined how policymakers and corporate houses will benefit from gauging the changing perceptions in the post-COVID world. They also explored the public sentiment about reopening in the new normal future [20]. Medford et al. applied topic modeling to show the sentiments about COVID-related topics, such as quarantine, transmission, and prevention, etc [21]. Paul presented an analysis on public sentiments over the opinions of social commentators and scientific communities about the origin of the virus [20]. It is imperative to suss the possible link between socioeconomic, geographical, political, and demographic factors of countries or US states and its public perception about adopting the emerging mitigation measures.

There have been efforts in leveraging network science to study the impact of public reservation over mask-wearing, vaccination, etc. Dinh et al. explored the relationship between the dynamics of virus and COVID information spread in social networks to show that they are interrelated but not identical [22]. Curiel compared the vaccination strategies against anti-vaccination views using diffusion models on different network topologies. They reported that reducing long distance trips or not allowing hubs (i.e., highly connected people in the social network) may dampen spread despite limited vaccine supply or anti-vaccination views [23]. Chung et al. explored the possibility that communication networks among health professionals can drive knowledge diffusion regarding COVID-19 mitigation [24]. Fagiolo studied the changing social network structure as more people are compelled to isolate due to the lockdown, linking disease diffusion and social network attributes [25]. Zhang et al. built a hidden multiplex network to quantify the interaction between information dissemination and virus spread [26].

### Contributions

In this work, we carry out a comprehensive analytical study on the tweets about COVID-19 to identify the relationship between public sentiment and the socioeconomic, demographic, political and epidemiological features of a given geographical region (i.e., both country and US state). We apply supervised machine (and deep) as well as unsupervised learning approaches to learn the opinions about the adoption of social distancing, masks, and vaccination as a means to curb pandemic spread. Our study reveals the overall absence of variation in public sentiment on the basis of spatial proximity or COVID-19 infection rates. Furthermore, we observe a reluctance to recognize vaccination as a COVID-19 mitigation strategy. Specifically, a time-series analysis suggests a gradual decline in positive sentiments on social distancing and vaccination, while an increase in confidence on the use of masks. Our regression analysis elucidates that the US states with (1) high age and ethnicity (but not gender) affect public sentiment in the US, and (2) busy airports having a cosmopolitan population seem more accepting of the scientific research about vaccination and mask-wearing, and (3) a larger homeless population are stronger advocates of mask-wearing.

## 2. Materials and Methods

Here, we discuss the dataset utilized in the study, machine learning models for sentiment analysis and the performance metrics.

### 2.1. Dataset

#### 2.1.1. COVID Tweet

We consider over 400,000 tweets between March to December 2020 from around the world. The tweets associated with COVID-19 were extracted in two steps. First, we referred to the repository that leveraged the Twitter’s streaming API to amass tweet IDs (see https://github.com/echen102/COVID-19-TweetIDs, accessed on 20 February 2020 and [27]). Specifically, this repository contains text files with IDs (corresponding to COVID-19 related tweets) organized into folders in monthly chronological order. Second, we approximately collected the tweets by utilizing the first two text files in each folder (corresponding to the first day of each month) in conjunction with the Python TweePy API [28]. Tweets that were not posted in the English language (35.22% of total tweets) were deleted after posting were not included in our dataset. Each tweet has the following fields: {*ID*, *date*, *tweet*, *location*}. We considered tweets that relate to the following three COVID-19 keywords, namely *mask*, *vaccine*, and *distancing*. A tweet is about a keyword if it contains any of the words mentioned below.

- **Mask.** mask, masking, mask-wearing, wear, covering, face
- **Vaccine.** vaccine, vaccination, immune, immunity, dose, injection, inoculate, shot
- **Distancing.** lockdown, distancing, distance, mobility, isolation, social distance, contact, quarantine

Evidently, a tweet can be linked with multiple keywords and will therefore be counted several times. The world tweet dataset is available on GitHub (https://github.com/satunr/COVID-19/blob/master/Sentiment/final_world.csv.zip, accessed on 20 February 2020).

#### 2.1.2. Latitude–Longitude Coordinates

The coordinates of the US states and countries are acquired from the Google Public Data Explorer [29]. It provides public data and forecasts (in a format called Dataset Publishing Language) in collaboration with several international organizations. The coordinates of the states and countries are shared in https://github.com/satunr/COVID-19/blob/master/Sentiment/Coordinate.xlsx, accessed on 20 February 2020.

#### 2.1.3. Population Counts

The population of US states and countries is shared in GitHub (see https://github.com/satunr/COVID-19/blob/master/Sentiment/Population.xlsx, accessed on 20 February 2020).

#### 2.1.4. Infection Counts

The infection counts for the US states are acquired from the data released by New York times (https://github.com/nytimes/covid-19-data, accessed on 20 February 2020). The infection count for the countries is taken from the COVID-19 Data Repository by the Center for Systems Science and Engineering at Johns Hopkins University (see https://github.com/owid/covid-19-data/blob/master/public/data/owid-covid-data.csv, accessed on 20 February 2020).

#### 2.1.5. Red vs. Blue States

We create lists of likely red and blue states as per the projection of the outcome of the 2020 US presidential elections from NPR news [30] (see https://github.com/satunr/COVID-19/blob/master/Sentiment/red-blue.txt, accessed on 20 February 2020).

#### 2.1.6. Demographic and Socioeconomic Features from the States of USA

We compiled this dataset from different open-access data portals. Each of the individual open-source datasets belonging to the 50 different US states and also the consolidated overall dataset are available on GitHub at the following link: https://github.com/satunr/COVID-19/tree/master/US-COVID-Dataset, accessed on 20 February 2020. We briefly explain the features compiled in this dataset below:

- *Age group* (total range: 0–80+ years) in groups of 4 years and also normalized them based on the population [31] (filename: source/Data_age.xlsx, feature name: age_to_, Norm_to_, e.g., age8to12 or Norm8to12); we have also grouped them in classes of 20 years later on for some of the analysis.
- *Percentage change in Gross Domestic Product* [32] It is measured in all 50 states and District of Columbia in 2020. (filename: source/GDP.xlsx, feature name: GDP).
- *Traffic or airport activity* quantifies the airport passenger traffic; it is also normalized by total airport traffic across all 50 US states [33] (filename: source/Data_airport.xlsx, feature name: Busy airport score, Normalized busy airport).
- *Gender* is the *male* and *female* fraction of the total population [34]
  (filename: source/Data_gender.csv, feature name: Male, Female).
- *Population density* is simply the ratio between the population of a state and its total area [31] (filename: source/Data_population.csv, feature name: Population, Area, Population Density).
- *Ethnicity* denotes the fraction(s) of total population that are *Asian*, *Hispanic*, *black*, and *white*; we did not consider the other smaller ethnicities [35]
  (filename: source/Data_ethnic.csv, feature name: Asian, Hispanic, Black and White).
- *Homeless* quantifies the total number of homeless individuals in each US state [36] (filename: source/Data_homeless.xlsx, feature name: Homeless). Similarly, for each state, the normalized homeless population designates the ratio between its homeless population and total population.
- *Healthcare index* is formally measured by the Agency for Healthcare Research and Quality (AHRQ). The quantification is done considering (1) the care setting (e.g., hospitals and nursing homes), (2) clinical area (e.g., caring for patients having diabetes, cancer), and (3) care type (e.g., chronic, preventive) [37] (filename: source/Data_health.xlsx, feature name: Health).

#### 2.1.7. Preprocessing

**Figure 1:**
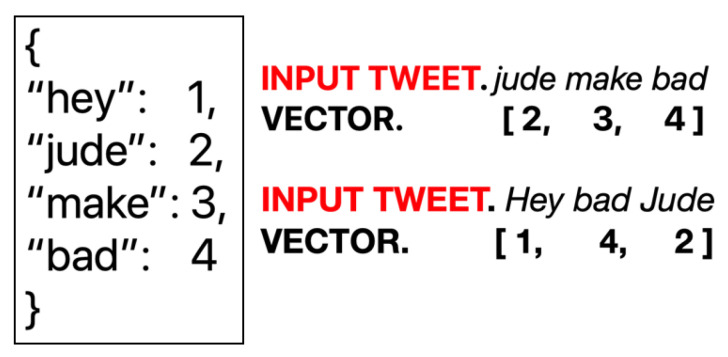
Vectorization of tweet data: Each token is assigned an ID, and each tweet is converted into a vector of token IDs.

We utilize the Python *Natural Language Toolkit* (NLTK) [38]—which is a standard library to process human language data—to acquire a labeled dataset of positive and negative tweets. In order to train a classifier, the labeled tweets are tokenized and stopwords are eliminated (Figure 1).

### 2.2. Sentiment Analysis Approaches

We discuss the machine learning approaches and performance metrics employed towards sentiment analysis.

#### 2.2.1. Machine Learning Approaches

- *K-fold cross validation.* It is a standard approach to test the efficacy of a classification model [39]. The labeled training dataset is split into *K* partitions. The classifier is trained on all possible K−1 partitions and tested on the *K*-th partition. The overall performance of the estimator is a mean of the accuracy of the predictions from the *K* folds. We used the Python *Scikit-learn library* [40] to perform cross-validation.
- *Naive Bayes (NB).* It is a class of fast, probabilistic learning techniques that apply the Bayes’ theorem to assign labels to the data points [41]. We use NB to learn the sentiments in an iterative approach. We train the NB classifier with the labeled NLTK tweet dataset and iteratively predict the label of p% unlabeled COVID tweet dataset. At each step, we apply *K*-fold cross validation (see Section 2.2.1) to monitor the accuracy of the integrated dataset.
- *Convolutional Neural Networks (CNN).* It is a class of deep learning which can assign varying importance to different aspects of the input data. This helps them distinguish these aspects from one another [42]. After the processing step (see Section 2.1.7) is complete, we assign a unique ID to the meaningful tokens and *vectorize* each labeled tweet as follows. Each labeled tweet is filtered to preserve *L* most common words and converted to a vector of token IDs (see Section 1), allowing us to preserve the relative order of the tokens. The CNN classifier is then trained on the (tweet vector, sentiment) pair and the accuracy, estimated by cross-validation, is recorded. At the end of the training stage, a tweet is associated with a label 2 or 0, representing positive or negative sentiments, respectively.
- *Multiple regression (MR).* Considering a dependent and independent variable *B* and *A* respectively, their linear association designated by the function B=g(A) is captured by the statistical measure of MR. The linear association is generated by MR in the following form: $\hat{B}=\beta \left(0\right)+\beta \left(f_{1}\right)A\left(f_{1}\right)+\beta \left(f_{2}\right)A\left(f_{2}\right)+\cdots +\epsilon$; here, ϵ is the error term, β(0) the intercept, and the coefficient $\beta (f_{i})$ quantifies how feature $f_{i}$ contributes to *b*, which is the dependent variable.
- *Curve fitting.* It is an approach that estimates the coefficients of a polynomial of degree *d* that minimizes the least squares error for input vectors *A* and *B* [43]. We utilize the Python NumPy library [44] to achieve curve-fitting.

CNN has been used to analyze image data, but also learn the relationship of text features in the absence of the knowledge of the entire context [45]. Like CNN, Naive Bayes has also emerged as a reliable approach for sentiment analysis. However, unlike CNN, Naive Bayes offers quick training with fewer parameters, yet it yields fairly accurate performance [46]. We use the curve-fitting to learn the evolving trends in the COVID-19 adoption rates, while the regression studies is intended to quantify the contributions of the several socioeconomic and demographic factors towards the adoption rates.

### 2.3. Performance Metrics

We also utilize the following metrics in our study.

#### 2.3.1. Accuracy

This metric is expressed as the ratio between the observations that were correctly predicted to the total number of observations [47]. In other words, in multilabel classification problems, it quantifies the fraction of agreements between the predicted labels and the actual ones:

(1) $$Acc=\frac{\mathrm{TN}+\mathrm{TP}}{\mathrm{TN}+\mathrm{TP}+\mathrm{FN}+\mathrm{FP}}$$

Here, TN, TP, FN, and FP respectively designate the true negatives, true positives, false negatives, and false positives.

#### 2.3.2. Euclidean Distance

It is the length of a line segment between any two given points *p* and *q* in a space of *m* dimensions. It is calculated as:

(2) $$D=\sqrt{\sum_{i}^{m}{\left(p_{i}-q_{i}\right)}^{2}}$$

#### 2.3.3. One Sample *t*-Test

This is a statistical testing that compares the mean of a sample data to a known value. T score captures the number of standard deviations by which the sample mean deviates from known value [48].

#### 2.3.4. Pearson Correlation Coefficient

This statistical metric captures the linear association between two different variables where −1 and 1 designate strong negative and positive correlation, respectively, and 0 designates that the variables that are not correlated. If *A* and *B* are the two variables, the Pearson correlation coefficient is given by $\frac{\mathrm{cov}(A,B)}{\sigma_{A}\times \sigma_{B}}$, where cov(A,B) and $\sigma_{A}$ denote the covariance matrix and standard deviations, respectively [49].

Accuracy has been employed to quantify the ability of the machine learning classifier to recognize the public sentiment. The Euclidean distance, in conjunction with a one-sample *t*-test and Pearson correlation coefficient, helps study the correlation between spatial proximity between places (states or nations) and similarity in public perceptions.

## 3. Results

This section comprises the following subsections: selection of classifier to learn tweet sentiments, effect of spatial proximity, infection rates and political ideology on sentiment, evolution of opinions about COVID mitigation measures, and the effect of socioeconomic, demographic, and epidemiological factors on sentiment.

### 3.1. Selection of Classifier

**Figure 2:**
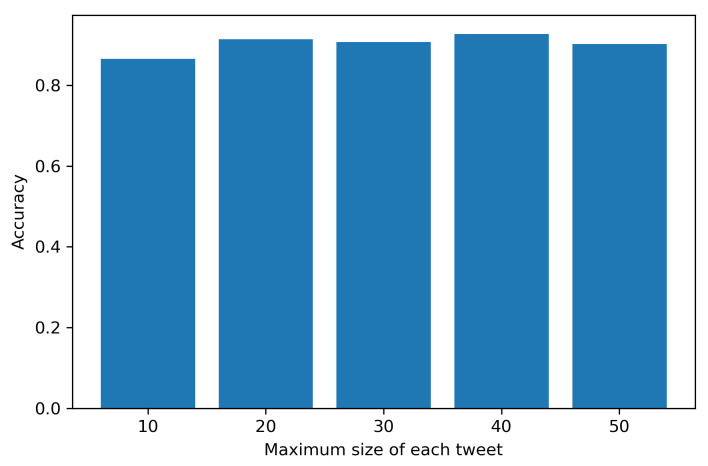
Accuracy of *K*-fold cross-validation on labeled NLTK tweet data using CNN with varying length of tweet vector.

We apply *K*-fold cross-validation on the Naive Bayes and CNN classifiers and determine their respective accuracy in predicting tweet sentiment. Figure 2 shows the varying cross-validation accuracy using CNN on the labeled NLTK tweet dataset (see Section 2.2 for details).

We vary the number of tokens in each tweet (also measured as the length of each tweet vector) L=10,20,⋯,50. However, the overall accuracy is approximately 85%.

**Figure 3:**
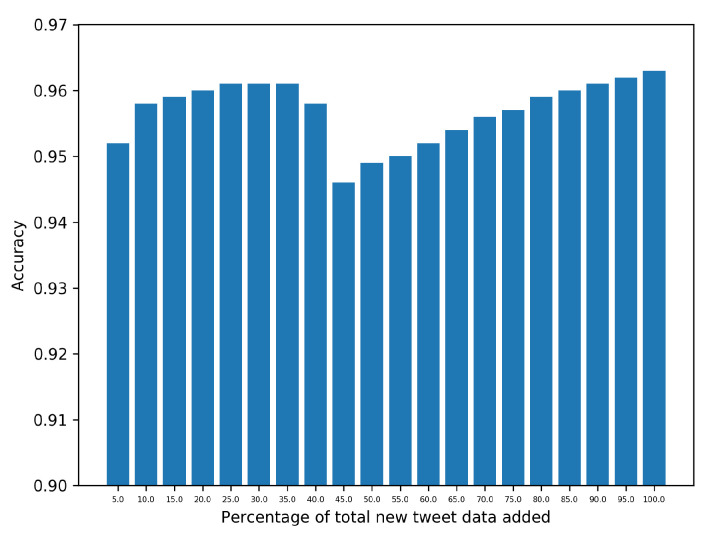
Accuracy of *K*-fold cross-validation (using Naive Bayes classifier) on augmented tweet data constituting labeled NLTK tweets and p=5%,10%,…, COVID-19 tweets.

A similar *K*-fold cross-validation experiment using the Naive Bayes (NB) classifier on the labeled NLTK tweet data yields an approximate sentiment prediction accuracy of 90%. As described in Section 2.2.1, we subsequently train NB with the augmented dataset (comprising labeled NLTK tweet dataset and part of COVID tweet data) and iteratively predict the label of p=5% yet *unlabeled* COVID tweets. Applying *K*-fold cross validation at each step, NB achieves an overall accuracy of around 95% on the combined dataset (as shown in Figure 3).

### 3.2. Correlation between Geographical Proximity and Sentiment

We utilize the Python library for geocoding, called GeoPy [50], to identify the latitude-longitude of the countries and US states. The details of this step are covered in Section 2.1.2. For each location (i.e., US state, country) *c*, we gauge the fraction of positive tweets in the region *F* (F>0.55). For any pair of regions $c_{i}$ and $c_{j}$, we calculate the Pearson’s coefficient (see Section 2.3.4) between the geographical proximity (measured in terms of Euclidean distance defined in Section 2.3.2) and the absolute difference between their fraction of positive tweets $F(c_{i})$ and $F(c_{j})$, i.e., $|F\left(c_{i}\right)-F\left(c_{j}\right)|$.

**Figure 4:**
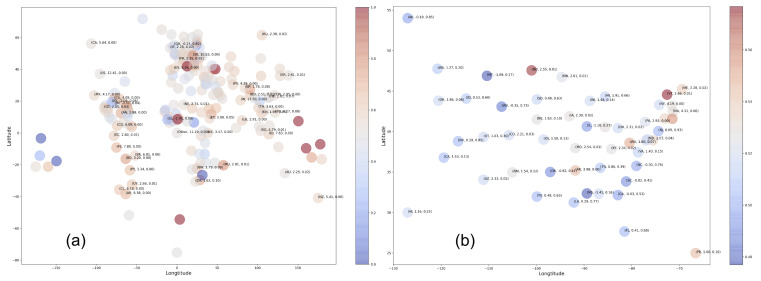
Visualization of the (**a**) countries and (**b**) US states based on latitude–longitude. The colors represent the fraction of positive tweets and annotations show (*t*-statistic, *p*-value) for hypothesis that the mean positivity in tweet sentiment is >0.5.

In Figure 4a,b, we place the countries and US states on their latitude–longitude, where the *F* is shown in different colors and annotated with the (*t*-statistic, *p*-value) for the hypothesis that the mean positivity in tweet sentiment is >0.5. The positive tweets and total tweets of the places with the least *p*-value and F>0.55 are summarized in Table 1. Note that countries like Azerbaijan, Bouvet Island, Gabon, Kyrgyzstan, Liechtenstein, Marshall Islands, Mauritius, Micronesia, Pitcairn Islands, Saint Vincent and the Grenadines, Solomon Islands, Togo, Tuvalu, and Vatican City that have F∼1.0 are not statistically significant as few tweets originate from these places. Pearson’s correlation, *p*-value, for world and US are $\left(0.03,2.3010^{-08}\right)$ and (−0.01,0.60), respectively, suggesting the overall lack of correlation between geographical proximity and public sentiment among the countries and US states.

### 3.3. Effect of Sentiments on COVID-19 Infection Rates

**Figure 5:**
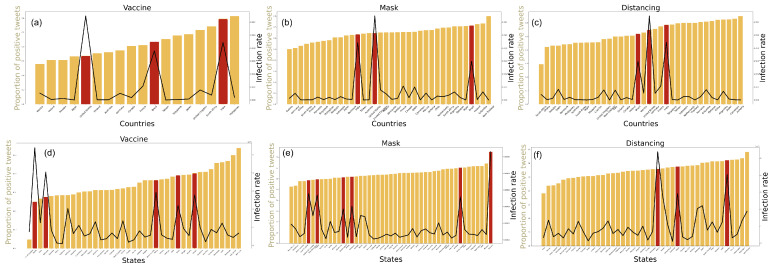
Fraction of positive tweets on vaccine, mask and social distancing from the (**a**–**c**) countries and (**d**–**f**) US states and their total infection numbers (between the tweet period) normalized by their population count.

We rank the countries and US states *c* in the increasing order of the positive sentiments F(c) corresponding to the tweet set on vaccine, mask, and social distancing. For each region (i.e., country and state) and tweet set, we calculate the total COVID-19 infection numbers spanning between the earliest tweet date of the tweet set and normalize it by the population of that place (see Section 2.1.3 for details). Figure 5a–f show the plots corresponding to vaccine, mask, and social distancing related tweets and infection numbers for the countries and states, respectively. The results show that USA, India, and Brazil (colored red) show a high normalized infection count, but they are spread all across the spectrum of countries ranked by sentiment. Similarly, Alaska, Delaware, and Maine (colored red) are amongst the states with the highest scaled infection count between the corresponding tweet dates. Interestingly, these states exhibit the least positive sentiment in case of vaccine (Figure 5d) and mask-wearing (Figure 5e), suggesting that the reluctance of the public in adopting COVID-19 mitigation measures may be a contributor to the overall contagion within the US. However, no such correlations can be established from the social distancing plot (Figure 5f).

### 3.4. Evolution of Public Sentiment over COVID-19

We isolate the tweets regarding (1) vaccine, (2) mask, and (3) social distancing related terms (refer Section 2.1.1 for the related terms of the three keywords). Since each tweet is associated with a date, we visualize the public sentiment (measured, once again, in terms of the fraction of positive tweets *F*) against the time-wise evolution of the three COVID-19 keywords.

**Figure 6:**
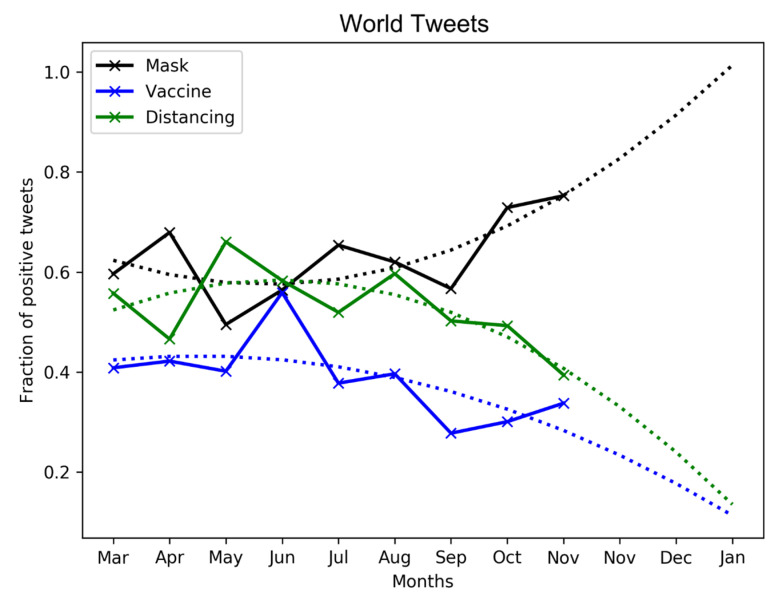
Evolution of public sentiment in world tweets about COVID-19 keywords, vaccine, mask, and distancing over time.

**Figure 7:**
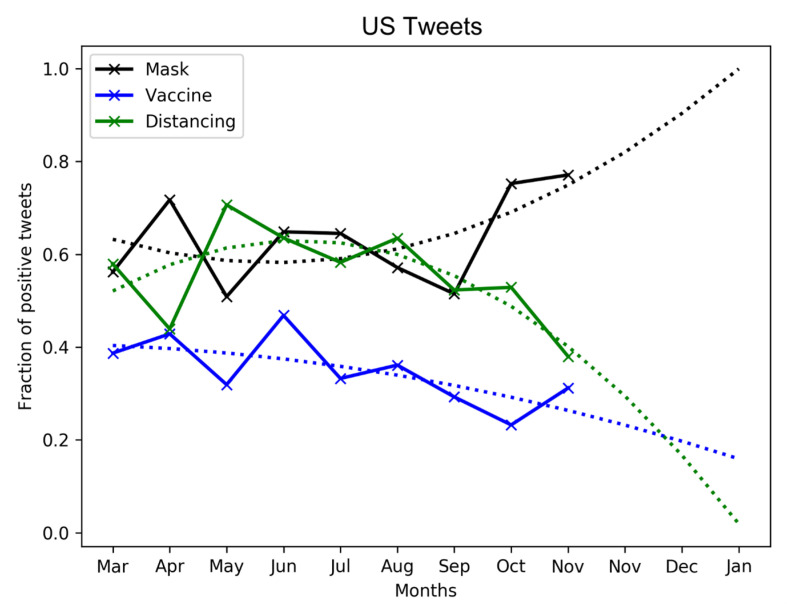
Evolution of public sentiment in US tweets about COVID-19 keywords, vaccine, mask, and distancing over time.

Figure 6 and Figure 7 show that there exist similar trends in public tweets from the world and US about vaccine, mask, and social distancing. In both cases, we see an increasing positivity over mask-wearing with F∼0.7 as of November 2020. We fit the curve to a polynomial of order 2 to show that the positivity in tweet sentiment over masking is expected to grow in 2021. Conversely, the trends are opposite in the case of vaccine and social distancing related tweets, as the adoption rates of these keywords continue to decline over time. It is evident that the netizens, mostly comprising a younger population, have either made light of the severity of the pandemic or been hesitant (a) to adopt social distancing and (b) about the efficacy of vaccines in combating pandemic spread.

### 3.5. Effect of Political Ideology on Sentiments

**Figure 8:**
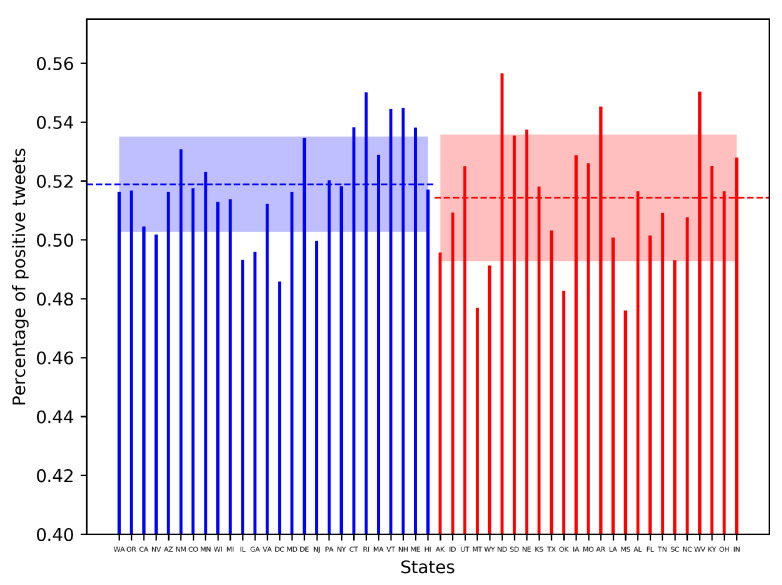
Mean and standard deviation in the fraction of positive sentiment tweets *F* for the blue and red states in USA.

We plot the fraction of positive tweets F(c) originating from each state *c* and estimate the mean and standard deviation in F(c) for the red and blue states (reported in Section 2.1.5). Figure 8 shows that F(c) for the red and blue states are comparable. The blue states tend to exhibit a marginally higher (and notably lesser variation in) positivity in COVID-19 related tweets.

### 3.6. Effect of Socioeconomic and Demographic Features on Sentiments on Adoption of COVID Mitigation Measures

We use multiple regression (see Section 2.2.1) to gauge the possible effect of the socioeconomic and demographic features of US states on the adoption rates of COVID-19 mitigation measures such as *vaccine*, *mask*, and *social distancing*. We consider a feature set comprising GDP, gender, ethnicity, health, normalized homeless, and three age brackets (0–30, 31–60 and >60) from the dataset (see Section 2.1.6 for details on each factor). For the labeled US tweet dataset, we consider the subsets of tweets containing (1) all terms, (2) vaccine, (3) mask, and (4) distancing related terms (refer to Section 2.1.1 for the related terms of the three keywords). Each feature value is normalized by the maximum value of that feature across all US states. For each state *c*, the dependent variable in the regression study is the fraction of positive tweets F(c) among the selected tweet subset.

**Figure 9:**
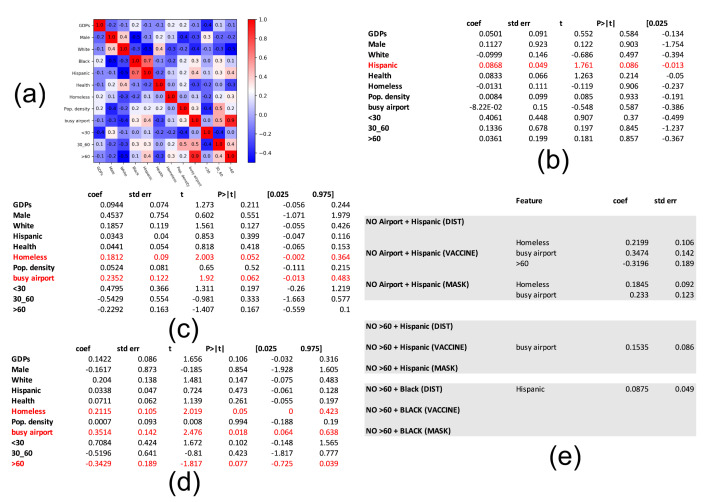
Regression analysis. (**a**) pairwise feature correlation; (**b**) complete regression table for feature set without airport and black features; significant features for regression of feature set without (**c**) airport and Hispanic) (**d**) age > 60 and hispanic, and (**e**) age >60, black.

Beginning with the feature set GDP, male, white, black, hispanic, health index, homeless, population density, airport traffic, and age groups <30, 30–60, >60, we measure the Pearson correlation between every pair of features. The features with a correlation equal to or in excess of 0.7 are not considered in the same regression analysis. Figure 9a shows two such pairs of correlated features, namely (age group >60, airport traffic) and (black, hispanic). Thus, in the regression analysis, we consider the four configurations without the following feature pairs: (1) (airport, black), (2) (airport, Hispanic), (3) (age >60, hispanic) and (4) (age >60, black), ensuring that the remaining features in each configuration are not highly correlated. We carry out the regression analysis for all four configurations and record the significant features (*p*-value <0.1).

We show the complete regression tables for social distancing, mask-wearing, and vaccination for configuration (1) in Figure 9b–d, respectively. Note that homeless and airport traffic emerge as the most significant features contributing positively to *F*. Hispanic and age >60 contribute positively and negatively to public perception around distancing and vaccination, respectively. Figure 9e shows the statistically significant features for configurations (2)–(4), where once again homeless and airport traffic emerge as the highest positive contributors. This suggests that the states with a high proportion of homeless are respectful of the effects of the pandemic and particularly how mask-wearing can mitigate its ill-effects. In addition, people living in states with high airport traffic, representing the cosmopolitan section of the US population, are clued in to the scientific advancements to curb spread. Similarly, the places with a relatively younger population (i.e., age ≤60) are flouting the COVID-19 related restrictions.

## 4. Discussion

While this study provides some valuable insights into the general attitude surrounding COVID-19 mitigation measures, it suffers from the usual challenges associated with any natural language processing based study. First, with regard to the sentiment analysis of the countries, the entire analysis is done for English tweets, which only accounts for 50% of global tweet dataset [51] in general and 64.78% of tweets in this study; therefore, it does not adequately represent the non-English speaking nations. However, we report the *p*-values in Section 3.2 to give the readers a sense of how reliable the sentiment for each country may be. Nonetheless, we find that the broad trends in sentiments are quite consistent across nations. Next, we show that the frequency of tweets across UK, USA vary greatly from countries like Togo and Solomon Islands, unavoidably skewing our measure of public perception. In addition, in many ways, it is more useful to know the sentiment of people hailing from smaller, developing nations. By the same token, the sentiments expressed by the netizens is not always the best representation of the worst-affected section of the society, such as the homeless or economically challenged. Finally, there is not enough data about the socioeconomic background of the individual issuing the tweet or his followers. This knowledge may present interesting understanding on the biases and the influence they may wield on public sentiment.

## 5. Conclusions

In this paper, we analyzed over 400,000 tweets from across the world to infer the evolution of public sentiments regarding the adoption of COVID-19 mitigation measures, namely vaccination, mask-wearing, and social distancing. We employ supervised machine learning methods in conjunction with natural language processing to identify the socioeconomic, demographic, epidemiological as well as political factors dictating public perception of the mitigation strategies.

Our analysis leads to several interesting findings. First, while there is a near-perfect balance between positive and negative sentiments globally, we see a lack of correlation between spatial proximity and public perception. Second, the blue states (in the USA) seem marginally more receptive to the COVID-19 mitigation measures, and the public at large seems to be responding positively to mask-wearing as a way of curbing spread. Third, our regression analysis reveals that the US states with a high number of homeless individuals show greater awareness of the perils of the pandemic and that high age and ethnicity (but not gender) are key predictors contributing to public attitudes about COVID-19.

## Figures and Tables

**Table 1:** Positive tweets, total tweets of countries and states with the least *p*-value and fraction of positive tweets F>0.55.

Place	Positive	Total Tweets
Argentina	359	520
Brazil	962	1651
India	4796	8051
Indonesia	794	1317
Peru	216	307
Guam	39	64
Vermont	169	293
North Dakota	166	289
Rhode Island	302	542
West Virginia	175	318

## Data Availability

The data and the codes are available online at https://github.com/satunr/COVID-19, accessed on 20 February 2020.
